# Neuroticism as a covariate of cognitive task performance in individuals with tinnitus

**DOI:** 10.3389/fpsyg.2022.906476

**Published:** 2022-08-02

**Authors:** Holly M. Edwards, James G. Jackson, Hannah Evans

**Affiliations:** School of Psychology and Therapeutic Studies, Faculty of Social and Health Sciences, Leeds Trinity University, Leeds, United Kingdom

**Keywords:** change blindness, cognitive performance, neuroticism, Stroop paradigm, tinnitus

## Abstract

Previous studies have shown cognitive task performance to be affected by tinnitus severity, but also that the literature is conflicted. This study sought to identify neuroticism as a possible confound, since severe tinnitus distress is associated with higher levels of neuroticism. A total of 78 participants (39 with and 39 without tinnitus) undertook two cognitive tasks. It was found that when undertaking a Stroop paradigm, controlling for neuroticism rendered previously significant results not significant. It was also found that neuroticism was not a significant covariate for a change blindness task. Gender, age, anxiety, and depression were all controlled for, and future implications for the literature discussed.

## Introduction

Tinnitus is the perception of sound, despite an absence of corresponding external acoustic stimulus (Durai and Searchfield, 2016). It is commonly described as a buzzing, whistling or ringing sound. It is estimated that between 10% and 15% of the population experience chronic tinnitus, while 1%–2% report significant reduction in quality of life and psychosocial wellbeing (Baguley et al., 2013). Tinnitus has high comorbidity with clinical depression, anxiety, insomnia, and is associated with concentration difficulties and emotional distress. Of note is the recent paper by De Ridder et al. (2021) who have suggested new operational definitions of tinnitus, distinguishing between ‘Tinnitus’ and ‘Tinnitus Disorder’—whereby the conscious awareness of sound is also accompanied by “emotional distress, cognitive dysfunction, and/or autonomic arousal, leading to behavioural changes and functional disability” (p. 1). Use of the latter definition, where appropriate, will enable recognition of tinnitus disorder as a condition in its own right.

There is evidence that tinnitus impacts on cognition in adults, either on its own or in combination with associated factors—e.g., anxiety and hearing loss (Tegg-Quinn et al., 2016). In a recent systematic review and meta-analysis of 38 studies, Clarke et al. (2020) suggested that subjective tinnitus interferes with executive function, general short-term memory, long-term storage/retrieval, and processing speed. That increasing tinnitus distress is associated with cognitive inefficiency, raises the possibility that cognitive tasks could assist researchers in distinguishing between patients with/without bothersome tinnitus or being used to objectively measure efficacy of tinnitus interventions over time.

There is growing evidence of tinnitus impinging on attentional resources, though explanations vary (e.g., Jackson et al., 2014). One theory is that increased tinnitus distress leads to an inability to ignore task-irrelevant information, leading to dual-task situations where participants attend to their tinnitus and to the task at hand, resulting in performance decrement. In contrast, individuals who have habituated successfully are better able to disregard their tinnitus, resulting in more typical performance. The distress associated with tinnitus (if any) is thought to be critical in moderating its effects on cognition (Leong et al., 2020) and as such, several authors have indicated that cognitive task performance could be an effective measure of tinnitus distress. For example, the Stroop paradigm is commonly used in tinnitus research. It does not utilize auditory resources and instead draws upon other modalities depleted through monitoring of the tinnitus sensation. Controlling for age, anxiety, and depression, Jackson et al. (2014) noted significantly lengthier reaction times in individuals with tinnitus over matched controls. In contrast, Waechter and Brännström (2015) found no main effect of tinnitus on task performance.

Neuroimaging studies have confirmed that limbic system and attentional resting state networks are more active in the presence of tinnitus and may explain why persistent tinnitus results in mental fatigue (Schmidt et al., 2013). Since chronic tinnitus generates consistent arousal, and since working memory is a finite resource, this suggests that working memory may be split between cognitive performance and tinnitus perception. While Waechter et al. (2020) failed to observe a moderating effect of tinnitus on working memory (WM), they did report near significant results and suggested “some potential for tinnitus to affect WM when the WM task is more difficult” (p. 5). Waechter et al. controlled for hearing threshold, anxiety and depression, warning that research into the effects of tinnitus on cognition has been constrained by limited consideration of confounding factors. Finally, Neff et al. (2021) looked at a clinical sample of tinnitus patients (*n* = 148), utilizing a novel machine-learning regression approach to consider the influence of tinnitus distress on a small battery of cognitive tasks. Interestingly, they concluded that task performance was clearly influenced by tinnitus distress.

One paradigm not yet considered in tinnitus research is Change Blindness (CB), defined as: “the striking inability to detect seemingly obvious changes that occur between views of a scene” (Murphy and Murphy, 2018, p. 655). Khan and Husain (2020) explain that tasks of selective attention fall into two broad categories—detection tasks and discrimination tasks. The Stroop paradigm is a discrimination task, requiring judgment of competing stimuli and consequently, greater use of cognitive resources. The Change Blindness Paradigm is a detection task, prompting identification of a target stimulus (i.e., a noticeable change). Higher-level processing is not required, whereas perceptual resources are. In a tinnitus context, it is important to consider which aspects of selective attention are being engaged by the tinnitus sensation. As Kahn and Husain state, if tinnitus engages perceptual resources, we can expect poorer performance on detection tasks. However, if tinnitus mostly utilizes cognitive resources, we should expect diminished performance in discrimination tasks. CB occurs when individuals do not notice changes to a scene with which they are actively engaged. Indeed, when changes are pointed out to the observer later, they express considerable surprise (Attwood et al., 2018). To interact effectively with the world around us, it is necessary to store visual perceptual information, and to perceive both stability and change. Failure to notice change is thought to be caused by an inability to note all objects in a complex scene and is increasingly common with age and higher perceptual load (Murphy and Murphy, 2018). CB can be measured by way of the Change Detection Flicker Paradigm (Bendall and Thompson, 2015), with two images alternated between in turn, identical but for a single change. Such tasks are easy unless the images are separated by a brief inter-stimulus interval (e.g., a grey screen). This interval masks sudden changes, forces more controlled and effortful searching, and is a robust and testable effect.

Persistent tinnitus has been shown to contribute to cognitive deficiencies, but there are comorbidities to consider, specifically anxiety and depression. In a review of 47 studies, Epp et al. (2012) revealed a substantial effect of clinical depression on the Stroop paradigm, the effect being so pronounced that the researchers suggested that further research into depression-related Stroop effects was “not necessary” (p. 316). Furthermore, Kalanthroff et al. (2016) have evidenced that high anxiety can affect Stroop task performance. Both anxiety and depression restrict working memory, selective attention, and executive function so it is essential that anxiety and depression are controlled for in tinnitus research.

However, it is most noteworthy that very limited consideration has been given to the effect of personality traits on task performance in tinnitus patients, though there has been recent focus on the relationship between tinnitus distress and neuroticism. Neuroticism is associated with perceived severity of tinnitus (Langguth et al., 2007), and may even be a risk factor in the development of chronic tinnitus (Holgers et al., 2005). In a cross-sectional study (*n* = 530), Bartels et al. (2010) found that individuals with tinnitus had significantly higher levels of neuroticism, social inhibition, and negative affectivity, as well as lower levels of emotional stability and extraversion, when compared with a control group. The authors concluded that personality tests could be capable of distinguishing between tinnitus and non-tinnitus individuals in a clinical setting. Utilizing 172,621 participants from the National Health Service register, McCormack et al. (2014), controlling for age, gender, and hearing loss, suggested that individuals scoring higher in neuroticism were more likely to report bothersome tinnitus. They concluded that personality traits, such as neuroticism, influence tinnitus severity through heightened sensitivity to intrusive experiences. This is supported by Simões et al. (2019) who measured the Big Five personality traits in 388 tinnitus patients attending a German outpatient clinic. Using a longitudinal design, the researchers investigated moderating effect of personality on patient outcome, concluding that neuroticism and extraversion “explain a large portion of the variance of tinnitus distress” (p. 6) in a clinical setting.

Currently, to the best of our knowledge, no study has measured the moderating effects of neuroticism on cognitive function in tinnitus patients. However, there is a great deal of research evidencing reduced cognitive efficiency in healthy adults who are high in neuroticism. It is suggested that such individuals are distracted by worry-related thoughts and that they are more readily aroused in stressful situations (Biernacki and Tarnowski, 2011). Further, Hahn et al. (2015) considered the effects of neuroticism on a change blindness task, noting that participants scoring highly on the neuroticism subscale of the Eysenck’s Personality Questionnaire Revised Short-Form (EPQR-S) noticed significantly fewer changes. Graham and Lachman (2012) used the backward digit span test to assess working memory in 4,947 adults and reported a significant negative correlation between neuroticism and number of digits recalled. However, age, gender, education level, hearing loss, and general health were all found to have an effect, making it difficult to conclude that neuroticism alone resulted in decreasing cognitive efficiency. Evidence that neuroticism affects selective attention is more limited. Utilizing the Stroop paradigm, Dreves (2017) saw a non-significant effect of neuroticism on reaction time. However, on administering a battery of cognitive tasks to older adults (*n* = 58), Williams et al. (2010) utilized the revised NEO personality inventory and evidenced that higher neuroticism scores were significantly related to reduced performance across all tasks.

Overall, the effect of tinnitus on cognition is unclear. Many individuals reporting tinnitus distress are likely to score highly on neuroticism scales, and neuroticism is not controlled for in tinnitus research. As such, neuroticism may be an experimental confound throughout the literature, contributing significantly to the current lack of clarity. The purpose of this paper is to further investigate the effects of the tinnitus sensation on cognitive efficiency, clarify whether tinnitus alone can affect cognitive efficiency, and whether neuroticism is a contributing factor to poor performance in a tinnitus sample. If so, cognitive task performance can only be considered useful in objectively assessing tinnitus distress if neuroticism is controlled for. Two aspects of cognitive ability will be measured: (i) a selective attention discrimination task (Stroop paradigm) and (ii) a visual working memory detection task (Change Blindness).

It is hypothesized that (1) Individuals with tinnitus will perform less well on at least one cognitive task than those without tinnitus, and this will take the form of slower reaction times, not increased error rates; (2) Neuroticism will be a significant covariate in both tasks; and (3) when Neuroticism is controlled for, there will be reduced differences in task performance.

## Materials and methods

This was a cross-sectional study making use of two cognitive tasks. Neuroticism (as measured by EPQR-S) was applied as a covariate where applicable. For the Stroop paradigm, 2 × 3 mixed ANCOVAs were utilized. The independent variable was Group (control/tinnitus), the repeated measure was type of stimulus (neutral/congruent/incongruent), with Neuroticism applied as a covariate if assumptions met. Dependent variables were response time (in milliseconds), and errors made. For the Change Blindness task, one-way ANCOVAs were utilized. The independent variable was Group (control/tinnitus) with Neuroticism applied as a covariate if assumptions met. Dependent variables were response time (in milliseconds), and errors made.

## Participants

A total of 78 nonclinical participants were recruited though convenience sampling in the United Kingdom, by way of public advertising, social media, and word of mouth. Participants with tinnitus had to confirm experience of constant ringing or buzzing (bilateral or unilateral) lasting longer than 3 months. Controls reported no noticeable tinnitus. All participants confirmed normal vision, or corrected-to-normal vision, that they were not color blind, and that English was their first language. No participants were unable to proceed but if this had occurred, participants were to be thanked for their time and debriefed accordingly. The sample consisted of 39 tinnitus volunteers and 39 controls without tinnitus (for Demographic Statistics, see Table 1). Assuming a large effect size (*f* = 0.40), *n* = 78, and an *α*-value of 0.05, G*Power v3.1 *post-hoc* calculations indicate achieved power of 0.882 for the 2 × 3 mixed ANCOVAs and 0.946 for the one-way ANCOVAs (Faul et al., 2009).

**Table 1:** Descriptive statistics for Stroop paradigm reaction times (ms).

Stroop stimulus	Group	Reaction time (ms)	Standard deviation
Neutral	Control	969.00	187.962
	Tinnitus	1063.37	273.324
Congruent	Control	942.21	179.242
	Tinnitus	1054.37	249.247
Incongruent	Control	1094.65	234.303
	Tinnitus	1283.02	402.475

## Materials

### Audiograms

To consider possible confounding effects of hearing loss, all participants were asked if they had issues with their hearing and to provide recent audiograms (last 12 months) where possible. A total of 30 participants self-reported possible hearing loss, including one control and 29 tinnitus patients. In total, 27 of the latter (69% of tinnitus sample) were able to provide a suitable audiogram, with mean dB loss calculated for the better hearing ear from hearing threshold values at 250, 500, 1,000, 2,000, 400, and 8,000 Hz. Mean hearing loss for tinnitus patients was 32.72 dB (SD 18.62). This suggests a tinnitus sample with mild hearing loss on average.

### Tinnitus functional index (TFI)

The TFI (Meikle et al., 2012) is a 25-item scale measuring tinnitus severity, higher scores indicating greater effect on daily functioning. Items include statements such as: “How often did your tinnitus make it difficult to FALL ASLEEP and STAY ASLEEP?” and “How BOTHERED or UPSET have you been because of your tinnitus?.” Each item is scored 0–10, giving a maximum score of 250, which is then converted to a score out of 100. It can be broken down into eight subscales: Intrusiveness, Sense of Control, Cognitive Interference, Sleep disturbance, Auditory Difficulties, Relaxation, Quality of Life, and Emotional Distress. Henry et al. (2016) suggests scores of <25 can be regarded as relatively mild with no need for intervention. Scores of 25–50 suggest significant difficulty and possible need of intervention. Scores >50 indicate severe tinnitus distress, and that intervention would be extremely beneficial. Fackrell et al. (2016) suggest that the TFI “measures the construct of tinnitus with excellent reliability in distinguishing between patients” (p. 220). They report excellent test–retest reliability for global scores (0.91) and subscales (0.81–0.95), with good internal consistency overall (Cronbach’s alpha (*α*) = 0.80).

### Hospital anxiety and depression scale (HADS)

HADS is a 14-item questionnaire designed to evaluate psychological distress in non-psychiatric patients. It has two 7-item subscales, namely, HADS-depression and HADS-anxiety. The former focuses on anhedonia (the inability to feel pleasure), whereas the latter focuses on symptoms of generalized anxiety disorder. Example items include “I still enjoy the things I used to enjoy” and “worrying thoughts go through my mind.” Each item is scored 0–3, giving a score of 0–21 for each subscale. Scores <8 can be regarded as “normal,” whereas scores of 8–10 are suggestive of possible dysfunction and are “borderline.” Scores of 11+ indicates probable presence of mood disorder (Bjelland et al., 2002). In their review of 15 studies, Bjelland et al. reported good internal consistency, with Cronbach alpha’s of 0.83 for anxiety and 0.82 for depression. Furthermore, HADS has been used in multiple studies associated with tinnitus distress (e.g., Andersson et al., 2013; Jackson et al., 2014).

## Eysenck personality questionnaire revised-short (EPQR-S)

The EPQR-S is a 48-item questionnaire assessing personality type. It has four subscales, but the present study made use of only the neuroticism and extraversion sub-scales (12 items each). The extraversion sub-scale was not analyzed, retained only to reduce response bias to neuroticism items—generally perceived by participants as being disadvantageous in nature. The EPQR-S neuroticism subscale measures the extent to which the individual is vigilant against potential harm, with high-scoring individuals more predisposed to anxiety and worry. All items were dichotomous (yes/no), giving a range of 0–12 for neuroticism. Example items include “Does your mood often go up and down?” and “Do you worry too long after an embarrassing incident?” Bartels et al. (2010) found good internal consistency for the neuroticism subscale (Cronbach’s *α* = 0.85) when using the EPQR-S to determine personality characteristics associated with tinnitus.

### Stroop paradigm

The task consisted of 108 items presented in lowercase Tahoma font (size 125); 36 congruent (e.g., the word ‘red’ written in red font), 36 incongruent (e.g., the word ‘red’ written in blue font), and 36 neutral (e.g., a line of four to six X’s presented in red font) trials. Furthermore, four colors were used (blue, green, red, and yellow), each appearing nine times in each type of trial. Participants were instructed to respond to the color of the font and ignore the word itself. An instruction screen was followed by 18 practice trials (six for each type of stimulus), with feedback (correct/incorrect). Any participant queries were answered, and then the task began. Stimuli were presented in the center of the screen in one randomized block, with a fixation cross (500 ms) prior to each trial. Each trial stimulus remained onscreen until the participant responded to the color of the font, pressing the corresponding button on the keyboard (1 = blue, 2 = green, 3 = red, and 4 = yellow). Throughout, a legend was present at the bottom of the screen, consisting of four colored boxes with the corresponding response key illustrated. On average, the task took 3 min to complete, and both reaction time and numbers of errors made were noted.

### Change blindness task

**Figure 1:**
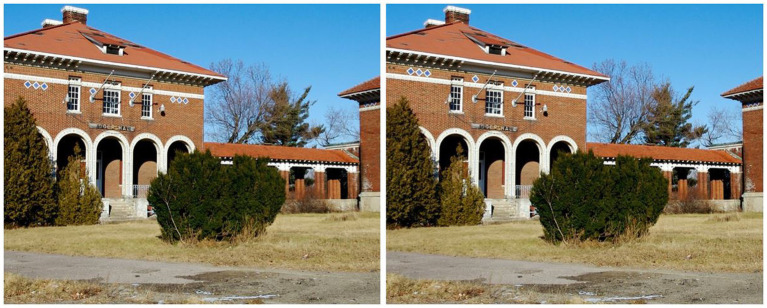
An example of image used in the change blindness task. Original image (left); Modified image (right) has diamond shapes above the white line, not below.

The change blindness task consisted of 15 pairs of digital photographs selected from a variety of natural scenes (e.g., indoor, wildlife, and city scenes) used in a previous experiment (Chau et al., 2011). Each original photograph was edited in Adobe Photoshop to generate a near-identical version with one discrete change (see Figure 1). As such, each pair of photographs was made up of one original image and one manipulated image. Five manipulated images had something added/removed, five had the location of an object changed, and five saw an object change color. Object location was balanced across all trials to minimize biases in the search strategies of participants. Subjects viewed these pairs in randomized order.

After preliminary instructions and two practice trials, participants were invited to press the spacebar and begin. A flicker sequence was initiated, with the original image (A) shown for 500 ms, followed by a grey buffer screen (blank) for 200 ms, then the modified image (B) was displayed for 500 ms, then the buffer again (200 ms), then the original image (A), and so on. As per Bendall and Thompson (2015), this use of a buffer screen masks sudden change and ensures the task is sufficiently challenging. Participants were instructed to visually search these alternating images until a change was detected, whereupon participants were to press the space bar. They would then be presented with four options, each corresponding to a number on the keyboard: (1) “An item in the two pictures changed color”; (2) “An item in the two pictures changed location”; (3) “An item in the two pictures appeared and disappeared”; (4) ‘I could not identify a change.” Participants chose one option to proceed to the next trial. Each trial lasted 60 s, and if allowed to complete, would show 50 images—25 (A) and 25 (B). If participants did not respond within this time limit, the trial ended with the participant only able to press (4) “I could not identify a change.” In the example (Figure 1), the correct response would be “2,” indicating a change of position. The task took an average of 10 min to complete and both reaction time and numbers of errors made were noted.

### Procedure

The experiment was run in a well-lit room on campus, utilizing COVID-secure guidelines as appropriate. Controls were asked to confirm they did not have tinnitus, viewing the TFI and stating the questions were not applicable. No control participants were excluded. Participants with tinnitus were asked to complete the TFI, before all participants were asked complete HADS and EPQR-S in counterbalanced order. Participants were then seated at the computer, undertaking both tasks in counterbalanced order. These were attempted in silence, with all stimuli presented by a 2.7GHz Dell computer with a 17″ display monitor, using the PsychoPy software package (Peirce et al., 2019), and presented in a screen resolution of 1,600 × 900. The whole study took approximately 20–25 min. No participants withdrew, and all were fully debriefed afterwards.

### Statistical analysis

Data was analyzed using quantitative methodology, with alpha values set at 0.05, and utilizing IBM SPSS v27. Shapiro–Wilk (*p* > 0.05 ns.) indicated normality. Main analyses were conducted utilizing Analysis of Variance (ANOVAs) and Analysis of Covariance (ANCOVAs) where appropriate, with Neuroticism (EPQR-S) as the covariate. Our primary analyses were: (1) Investigation of potential confounding factors (i.e., age, anxiety, depression, and neuroticism); (2) Evidencing whether tinnitus affected cognitive task performance; and (3) Evidencing whether tinnitus affected cognitive task performance when controlling for Neuroticism. Effect sizes were calculated as partial eta-squared, and targeted *post hoc* analyses (not corrected for pairwise comparisons) were conducted with *t*-tests. For both cognitive tasks, reaction time and error rates were considered.

## Results

### Questionnaires

#### Tinnitus functional index

Tinnitus participants scored 17.35 (SD 22.04) on the TFI, indicating a less distressed sample with relatively mild tinnitus (Henry et al., 2016). A total of 14 participants had a score <25, 16 between 25 and 50, and nine >50 (indicating severe tinnitus).

#### Hospital anxiety and depression scale

For the HADS-anxiety subscale, mean scores were 5.56 (SD 3.226) for controls and 6.95 (SD 3.471) for tinnitus participants. A total of eight participants self-reported anxiety ≥11, three of which were in the control group. There was no main effect of group on anxiety [*F* (1, 76) = 3.329, *p* = 0.072 ns.; partial eta squared = 0.042]. For HADS-depression, means were 3.28 (SD 2.635) for controls and 2.97 (SD 2.497) for individuals with tinnitus. Data was not normally distributed, so a Kruskal–Wallis ANOVA saw no main effect of group depression [H (1) = 0.106; *p* = 0.745 ns.]. One participant from the tinnitus group self-reported probable depression ≥11.

#### Eysenck personality questionnaire revised-short

Controls reported a mean neuroticism score of 4.08 (2.709) while those with tinnitus reported 5.38 (2.662). Data was normally distributed for both groups. An univariate ANOVA indicated a significant main effect of group membership [*F* (1, 76) = 4.625, *p* = 0.035; partial eta squared = 0.057], with the tinnitus group significantly higher in self-reported neuroticism.

### Tasks

#### Stroop paradigm (reaction time)

A 2 × 3 mixed ANOVA was utilized to investigate possible effects of tinnitus on reaction time. The independent variable was group (control/tinnitus) and the repeated measure was type of stimulus (neutral/congruent/incongruent). The dependent variable was reaction time (in milliseconds) for correct responses. All data was normally distributed, and descriptives can be found in Table 1.

There was a significant main effect of stimulus [*F* (2, 152) = 60.207; *p* = 0.000; partial eta squared = 0.442], with Bonferroni *post-hoc* tests indicating significantly increased latency for incongruent stimuli in comparison with neutral (*p* = 0.000) and congruent (*p* = 0.000) stimuli. There were no differences between neutral and congruent stimuli (*p* = 0.214 ns.). These results confirm the presence of the Stroop effect. There was no significant main effect of group [*F* (1, 75) = 3.662; *p* = 0.059 ns.; partial-eta squared = 0.047], though this result tended towards significance. There was a significant interaction between group and stimulus [*F* (2, 152) = 3.390, *p* = 0.036; partial eta squared = 0.043].

To consider Hypotheses 2 and 3, the same analysis was repeated controlling for neuroticism. In running this 2 ×3 mixed ANCOVA (with neuroticism as the covariate), no ANCOVA assumptions were violated, and data was normally distributed. See Table 2.

**Table 2:** Descriptive statistics (adjusted means) after controlling for neuroticism.

Stroop stimulus	Group	Reaction time (ms)	Standard error
Neutral	Control	993.53	34.662
	Tinnitus	1038.84	
Congruent	Control	963.97	32.374
	Tinnitus	1032.62	
Incongruent	Control	1136.65	45.899
	Tinnitus	1241.02	

**Figure 2:**
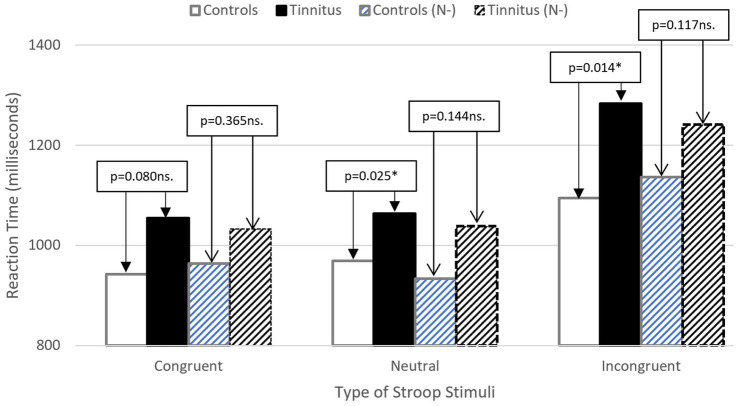
Illustrating reduced difference between groups for stimulus reactions time after controlling for Neuroticism (N-). Patterned bars indicate adjusted means (stroop RT).

The covariate, neuroticism, was significantly related to reaction time [*F* (1, 75) = 23.797; *p* = 0.000; partial eta squared = 0.241]. Controlling for neuroticism saw no significant main effect of group [*F* (1, 75) = 2.087; *p* = 0.153 ns.; partial eta squared = 0.027], no significant main effect of type of stimulus time [*F* (2, 150) = 1.015; *p* = 0.365 ns.; partial eta squared = 0.013], and no significant interaction [*F* (2, 150) = 1.303; *p* = 0.275 ns.; partial eta squared = 0.017]. The effect of tinnitus, after controlling for neuroticism is considered in Figure 2.

#### Stroop paradigm (error rate)

Due to low error rates, only total number of errors were considered. Data was not normally distributed for either group (Shapiro–Wilk < 0.05), and normality was not reconciled by removal of outliers. While controls made 0.82 errors (SD 1.167) and participants with tinnitus made 1.28 errors (SD 1.746). A Kruskal–Wallis ANOVA indicated no significant effect of group on total number of errors made [*H* (1) = 1.365; *p* = 0.243 ns.]. Assumptions were violated, so it was not appropriate to make use of neuroticism as a covariate.

#### Change blindness task (reaction time)

**Figure 3:**
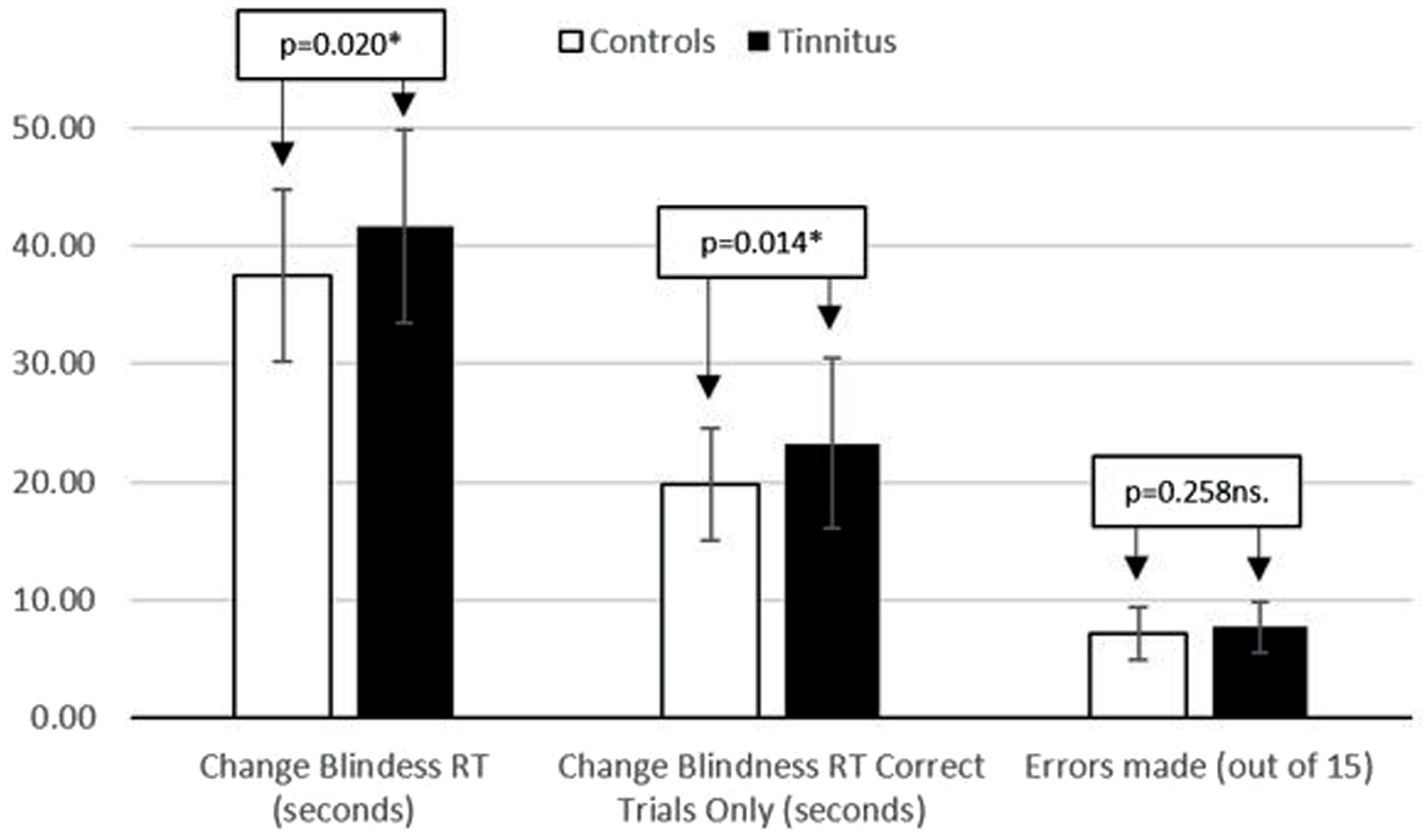
Displaying reaction time (seconds) and number of errors made during the change bilndness task.

A one-way univariate ANOVA was utilized. The independent variable was group (control/tinnitus) and the dependent variable was reaction time (seconds) taken to spot the difference between two images and respond correctly. All data was normally distributed (see Table 3), and comparisons of group performance can be found in Figure 3.

**Table 3:** Change blindness task descriptives, considering RT, and errors made.

Group	Change blindness RT (all trials)	Change blindness RT (correct trials)	Errors made
Control	37.44 (7.276)	19.80 (4.803)	7.15 (2.195)
Tinnitus	41.61 (8.192)	23.27 (7.210)	7.72 (2.176)
Overall	39.52 (7.978)	21.53 (6.333)	7.44 (2.190)

SDs in brackets.

There was a significant main effect of group on change blindness RT for all trials [*F* (1, 76) = 5.646; *p* = 0.020; partial eta squared = 0.069] and when only considering correct trials [*F* (1, 76) = 6.294; *p* = 0.014; partial eta squared = 0.076]. Those with tinnitus responded significantly more slowly. A one-way ANCOVA was run, adding neuroticism as a covariate. Data remained normally distributed and no ANCOVA assumptions were violated. The covariate, neuroticism, was not significantly related to reaction time [*F* (1, 75) = 0.136; *p* = 0.713 ns.; partial eta squared = 0.002], and the significant main effect of group remained [*F* (1, 75) = 6.301; *p* = 0.014; partial eta squared = 0.077].

#### Change blindness task (error rate)

A one-way univariate ANOVA was utilized, with the independent variable being group (control/tinnitus) and the dependent variable being error rate, as defined by a wrong answer, or failure to spot the difference between two images within a minute. All data was normally distributed, with descriptives in Table 3. There was no significant main effect of group [*F* (1, 76) = 1.299; *p* = 0.258 ns.; partial eta squared = 0.017]. When neuroticism was added as a covariate, no assumptions were violated. However, neuroticism, was not related to error rate [*F* (1, 75) = 1.994; *p* = 0.162 ns.; partial eta squared = 0.026], and there was no main effect of group [*F* (1, 75) = 0.632; *p* = 0.440 ns.; partial eta squared = 0.008].

## Discussion

Results from the Stroop paradigm resulted in all three hypotheses being accepted. As supported by previous literature (e.g., Jackson et al., 2014), the presence of tinnitus resulted in significantly slower response times for incongruent and congruent Stroop stimuli, suggesting that cognitive performance could be an effective measure of tinnitus distress. However, once neuroticism was controlled for, these significant differences disappeared (*p* > 0.05 ns.). This may be one possible explanation for the inconsistency of findings within the tinnitus literature, namely that differences in Stoop task performance can be explained by the confounding presence of neuroticism, which tends to be higher in the presence of more severe tinnitus distress McCormack et al. (2014). In this study, the tinnitus sample was significantly higher in neuroticism than a matched control group and performed significantly less well with regards to reaction time. When unmoderated by neuroticism, presence of tinnitus had no effect on Stroop error rate (Clarke et al., 2020). Noting the recent suggestion by De Ridder et al. (2021) that presence of ‘Tinnitus’ should be distinct from any associated “Tinnitus Disorder,” it is worth reiterating that merely having tinnitus is distinct from making a subconscious and emotional decision to orientate towards the tinnitus sensation as a perceived threat. In the case of the latter, higher levels of neuroticism may encourage this orientation and reduce the level of cognitive resources available for tasks at hand.

With regards to the change blindness task, only Hypothesis 1 can be accepted. It would appear that this selective attention (detection) task is affected by the presence of tinnitus, but that performance is not affected by neuroticism (Hypotheses 2 and 3). As with Stroop, the presence of tinnitus reduces reaction time, but does not increase errors made. This supports previous theories of cognitive inefficiency (e.g., Jackson et al., 2014), though goes against findings of Hahn et al. (2015) who reported that higher neuroticism scores predicted a reduction in task performance on change blindness tasks. However, Hahn et al. also reported considerably higher neuroticism scores than those in the present study.

Neuroticism is associated with the tendency to perceive surroundings as being threatening and difficult to manage (Slavish et al., 2018). Furthermore, neuroticism can lead to reduced performance in public and/or when being appraised (Uziel, 2015). To undertake novel cognitive tasks under laboratory settings is the very definition of appraisal and may result in unpredictable results when participants have tinnitus—especially with severe tinnitus distress and higher neuroticism scores. It may be that type of task is important, with abstract tasks such as the Stroop paradigm being more vulnerable to moderation by neuroticism than those which are more familiar—i.e. the Change Blindness Task is similar to the “Odd One Out” puzzles of childhood. Indeed, Schneider et al. (2012) highlights that neuroticism is known to “uniquely” (p. 106) impair task performance in unfamiliar situations, due to increased threat appraisal (e.g., negative judgments of others). Furthermore, this reduced performance on a detection task suggests that tinnitus engages perceptual resources rather than higher-order cognitive resources (Khan and Husain, 2020). As such, when introducing tasks to consider cognitive performance in tinnitus patients, great care should be taken to (i) make use of familiar tasks and/or ensure a substantial induction process and (ii) consider detection tasks of selective attention rather than discriminatory tasks.

It should also be highlighted that the change blindness task was not easy, with a near 50% error rate. While there were significant differences in RT across all trials (see Figure 3), this also includes those trials where the 60-s timer ran out. As such, correct trials RT is a more representative DV but these response times were based on an average of 7–8 responses and need to be considered with caution. However, the change blindness task was very demanding, requiring 10–12 min of sustained effort. There is a practical trade off to be considered between number of trials and overall length of task.

In this study, neither controls nor tinnitus participants differed in terms of age, or self-reported anxiety/depression (as measured by HADS). Although no participants expressed difficulty in using the keyboard, previous studies have shown that computer familiarity could be a factor (e.g., Iverson et al., 2009). To mitigate this, future studies may want to consider verbal responses. Finally, this study did not consider provide audiometric information for all participants. Certainly, the control group was not aware of any hearing loss, but this does not mean that no hearing loss existed. Unfortunately, due in part to Coronavirus restrictions, experimenter provision of hearing tests was not possible. With greater reason to have previously obtained a hearing test, the tinnitus group reported a mean hearing loss of 32.72 dB. This is not an unexpected finding as chronic tinnitus is frequently accompanied by hearing impairment (Jafari et al., 2019), and in a review of 11 other studies, Yuan et al. (2018) suggested that hearing-impaired participants were at higher risk of long-term cognitive impairment than normal hearing counterparts. However, it is to be noted that these studies made use of significantly older populations, and that when controlling for hearing loss, Waechter et al. (2020) found no difference in cognitive performance between their control group and those with tinnitus. In this study, it would appear that our probable difference in hearing threshold between groups did not affect anxiety or depression, which would themselves affect cognitive task performance. It is worth considering whether hearing loss is the reason behind the different levels of neuroticism between the groups. In a recent study, Nordvik et al. (2021) note that while neuroticism has no effect on hearing loss, it is associated with worse (self-reported) outcomes for hearing disability. The authors also note that at least 50% of the variability in neuroticism is determined genetically. It is also known that neuroticism tends to decease over the lifespan but as a trait measure, these changes are across decades, and it is not likely that study participants saw increases in neuroticism after tinnitus onset. In addition, this was a nonclinical convenience sample, and TFI scores were lower than is usually seen in the literature. As such, taken on their own, it is not advised that these results are generalized across the tinnitus population as a whole.

## Conclusion

The present study documents the confounding effects of neuroticism on cognitive task performance in individuals with tinnitus. This study does not imply that cognitive task performance could not be an effective objective measure of tinnitus distress, but researchers should acknowledge that individual differences such as trait neuroticism can be confounding factors—and future studies must be designed accordingly. If neuroticism is not controlled for, substantial experimental induction and practice elements are essential when making use of tinnitus participants under laboratory conditions. Furthermore, if the literature choses to distinguish between presence of tinnitus and “tinnitus disorder” as a medical condition (see De Ridder et al., 2021), it becomes of critical importance to disentangle neuroticism and associated threat appraisal from self-reported tinnitus distress. Finally, it is noted that as a detection task, the change blindness paradigm may be more appropriate than the Stroop paradigm (discrimination task), and this is also worthy of further research.

## Data availability statement

The raw data supporting the conclusions of this article will be made available by the authors, without undue reservation.

## Ethics statement

The studies involving human participants were reviewed and approved by Leeds Trinity University School of Social and Health Sciences Ethics Panel. The patients/participants provided their written informed consent to participate in this study.

## Author contributions

HME: methodology, software, formal analysis, investigation, writing—initial draft, and writing—review and editing. JJ: conceptualization, methodology, software, formal analysis, investigation, resources, data curation, writing—initial draft, writing—review and editing, visualization, supervision, project administration, and funding acquisition. HE: validation and writing—review and editing. All authors contributed to the article and approved the submitted version.

## Funding

This work was supported by the British Tinnitus Association Small Grant Scheme (£388); funding recipient James Jackson, Leeds Trinity University, United Kingdom.

## Acknowledgments

We would like to thank Kari Hoffman (provision of images) and Michelle Dalton for their assistance in making this research possible.

## Conflict of interest

The authors declare that the research was conducted in the absence of any commercial or financial relationships that could be construed as a potential conflict of interest.

## Publisher’s note

All claims expressed in this article are solely those of the authors and do not necessarily represent those of their affiliated organizations, or those of the publisher, the editors and the reviewers. Any product that may be evaluated in this article, or claim that may be made by its manufacturer, is not guaranteed or endorsed by the publisher.
